# Supplementary material for: Predicting visual function by interpreting a neuronal wiring diagram
Source: Nature. 2024 Oct 2;634(8032):113–23. doi: 10.1038/s41586-024-07953-5 (PMC11446822; doi:10.1038/s41586-024-07953-5)
Supplement: Supplementary file 6 — CRF and ERF predictions for individual TmY4 and TmY9 cells. Analogous to Supplementary Data 3, but for TmY target types. Shown are the top four monosynaptic pathways, the strongest pathway passing through each of the top ten intermediary types (ranking from Extended Data Fig. 7), and the trisynaptic pathway Tm1–TmY–Dm3–TmY (see the section entitled Prediction of spatial normalization). [file 41586_2024_7953_MOESM6_ESM.zip › DataS4/TmY9qΓèÑ/720575940618964225.pdf]

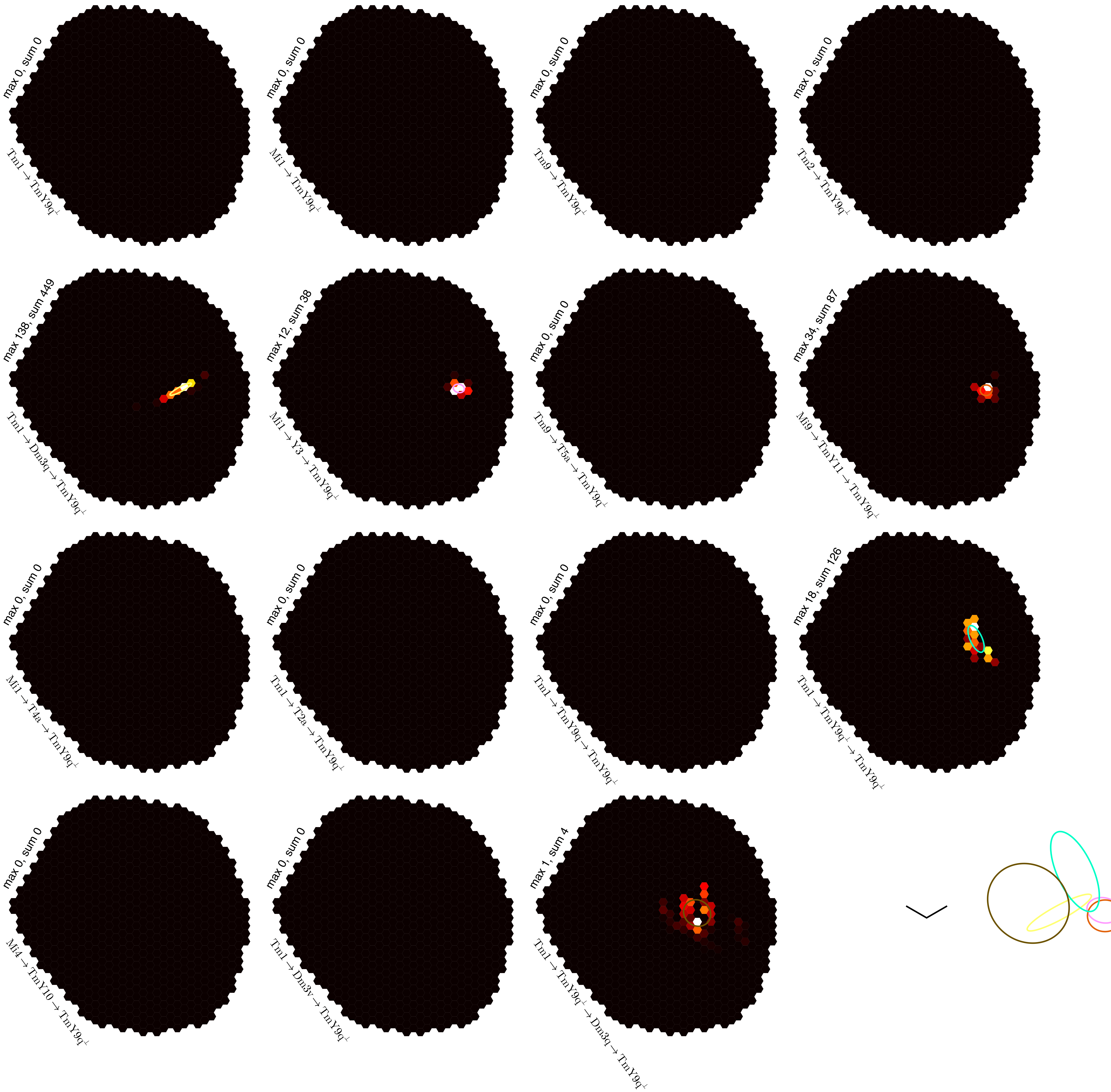
max 0, sum 0
Tm1 → TmY9q⊥
max 0, sum 0
Mi1 → TmY9q⊥
max 0, sum 0
Tm9 → TmY9q⊥
max 0, sum 0
Tm2 → TmY9q⊥
max 138, sum 449
Tm1 → Dm3q → TmY9q⊥
max 12, sum 38
Mi1 → Y3 → TmY9q⊥
max 0, sum 0
Tm9 → T5a → TmY9q⊥
max 34, sum 87
Mi9 → TmY11 → TmY9q⊥
max 0, sum 0
Mi1 → T4a → TmY9q⊥
max 0, sum 0
Tm1 → T2a → TmY9q⊥
max 0, sum 0
Tm1 → TmY9q → TmY9q⊥
max 18, sum 126
Tm1 → TmY9q⊥ → TmY9q⊥
max 0, sum 0
Mi4 → TmY10 → TmY9q⊥
max 0, sum 0
Tm1 → Dm3v → TmY9q⊥
max 1, sum 4
Tm1 → TmY9q⊥ → Dm3q → TmY9q⊥
